# Supplementary material for: Vacuum ultraviolet spectroscopy of the lowest-lying electronic state in subcritical and supercritical water
Source: Nat Commun. 2017 May 17;8:15435. doi: 10.1038/ncomms15435 (PMC5442368; doi:10.1038/ncomms15435)
Supplement: Supplementary Information — Supplementary Figures, Supplementary Tables, Supplementary Notes and Supplementary References [file ncomms15435-s1.pdf]

	i	23	100	150	200	250	300	350
		Temperature (°C)						
A (M^{-1} cm^{-1})	0	1480	1387.4	1385.8	1383.7	1347.9	1264.6	1157.1
	1	380.03	476.69	463.77	465.46	475.82	384.25	389.53
	2	372.67	320.19	273.82	161.66	99.125	32.999	96.569
	3*	4.2458	4.3574	4.4348	4.1415	4.1765	4.0032	3.8847
E_{max} (eV)	0	8.5501	8.3990	8.2844	8.1503	8.0715	7.9217	7.9285
	1	7.8080	7.6753	7.5851	7.5079	7.4692	7.3866	7.4469
	2	8.1191	7.9793	7.8762	7.7951	7.7391	7.6055	7.2981
	3	7.5454	7.3987	7.3578	7.1489	7.0770	6.9249	6.8209
σ (eV)	0	0.56305	0.56151	0.55547	0.55605	0.55747	0.53832	0.54789
	1	0.31593	0.32091	0.31789	0.30715	0.32608	0.32418	0.37064
	2	0.34329	0.32891	0.29013	0.23563	0.20514	0.17898	0.27526
	3	2.0526	2.0538	2.0838	2.0404	2.0454	2.0187	1.98109
cutoff (Å)		1440	1470	1480	1500	1520	1540	1550

Supplementary Table 1 | Fitted parameters for subcritical water VUV and near-UV absorption data. Fitted parameters for the 4-Gaussian fit to subcritical VUV combined with energy shifted near-UV data of Kröckel and Schmidt[1] are shown at all experimental temperatures. Fitting parameters are defined in equation 1. The cutoff wavelength indicates the lowest wavelength at which the fit is reliable.

*Coefficient A_3 must be multiplied by 10^{-5} to fit the very small extinction near-UV tail of the spectrum.

Pressure (bar)	Density (g cm^{-3})	Fractions				
		Monomer			Dimer	Trimer
		Unperturbed	Perturbed	Total		
4	1.33×10^{-3}	0.960	0.0319	0.992	8.35×10^{-3}	1.33×10^{-4}
17	5.78×10^{-3}	0.837	0.127	0.964	0.0335	2.21×10^{-3}
36	0.0126	0.679	0.247	0.926	0.0655	8.75×10^{-3}
65	0.0240	0.483	0.388	0.871	0.105	0.0238
98	0.0390	0.314	0.503	0.817	0.139	0.0445
139	0.0621	0.166	0.593	0.759	0.170	0.0718
170	0.0856	0.088	0.633	0.721	0.187	0.0921
200	0.120	0.0366	0.651	0.688	0.201	0.111
219	0.156	0.0144	0.655	0.669	0.208	0.122
229	0.192	5.92×10^{-3}	0.654	0.660	0.212	0.128
237	0.265	9.93×10^{-4}	0.652	0.653	0.214	0.133
245	0.402	3.40×10^{-5}	0.646	0.646	0.217	0.138
263	0.476	5.38×10^{-6}	0.631	0.631	0.222	0.148
280	0.505	2.54×10^{-6}	0.617	0.617	0.226	0.157
300	0.529	1.41×10^{-6}	0.602	0.602	0.231	0.167

Supplementary Table 2 | Experimental SCW pressures and densities, and corresponding monomer, dimer, and trimer fractions. The experimental pressures and densities for SCW spectra acquired at 381 °C are listed, along with known associated fractions of total monomer, dimer, and trimer species, as calculated from Tretyakov, *et al.*[2] Perturbed and unperturbed monomer fractions are calculated from the spectral fits as described in the text.

i	A (M^{-1} cm^{-1})	E_{max} (eV)	σ (eV)
0	335.21	7.0853	0.18775
1	88.167	7.2426	0.09585
2	111.08	6.9809	0.10795
3	455.81	7.3483	0.24849
4	926.83	7.6103	0.47809

Supplementary Table 3 | Fitting parameters for low-density 381-°C SCW spectrum. We list the fitting parameters for 5-Gaussian fit to 4-bar SCW unperturbed monomer spectrum at 381 °C, which largely reproduce the water gas-phase spectrum. Fitting parameters are defined in equation 1.

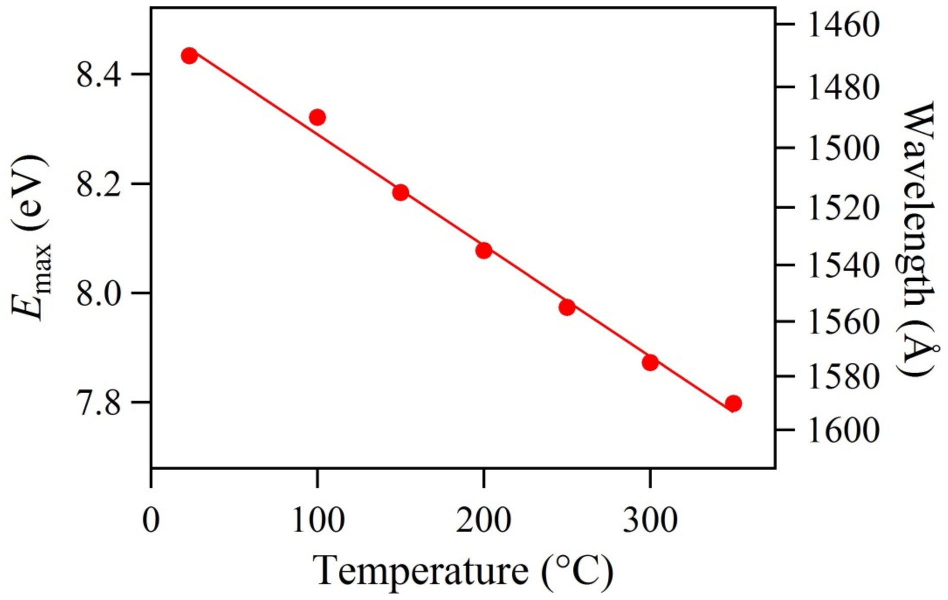

Supplementary Figure 1 | Temperature dependence of subcritical water VUV energy of maximum absorption. The observed shift in the water VUV maximum energy of absorption E_{max} and maximum wavelength of absorption are shown as a function of temperature, demonstrating a nearly linear dependence of the absorption maximum on temperature, with a shift of -0.00203 eV °C^{-1}.

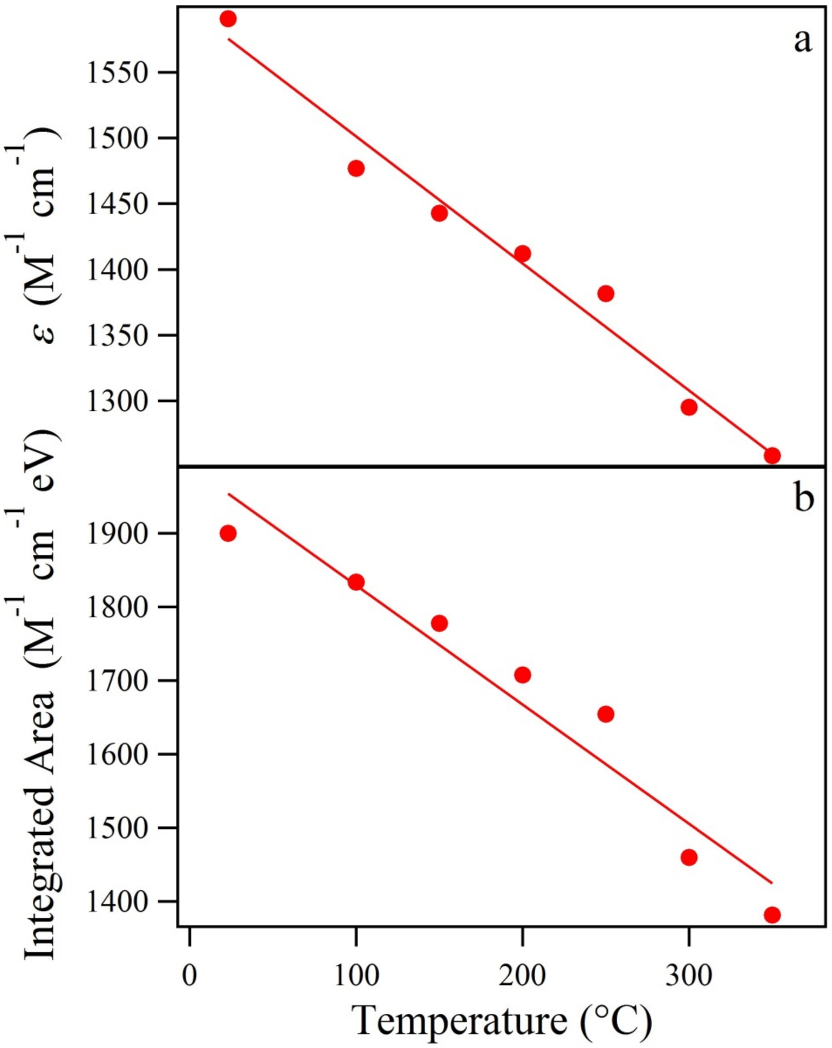

Supplementary Figure 2 | Temperature dependence of subcritical water maximum extinction coefficient and integrated absorption area. A gradual decrease in the maximum extinction coefficient ε of subcritical water is observed with increasing temperature (**a**) as the spectrum red shifts and broadens. The integrated area under the absorption curve shows a similar decrease with increasing temperature (**b**), which we suggest may be account for by changes in the water refractive index as a function of temperature.

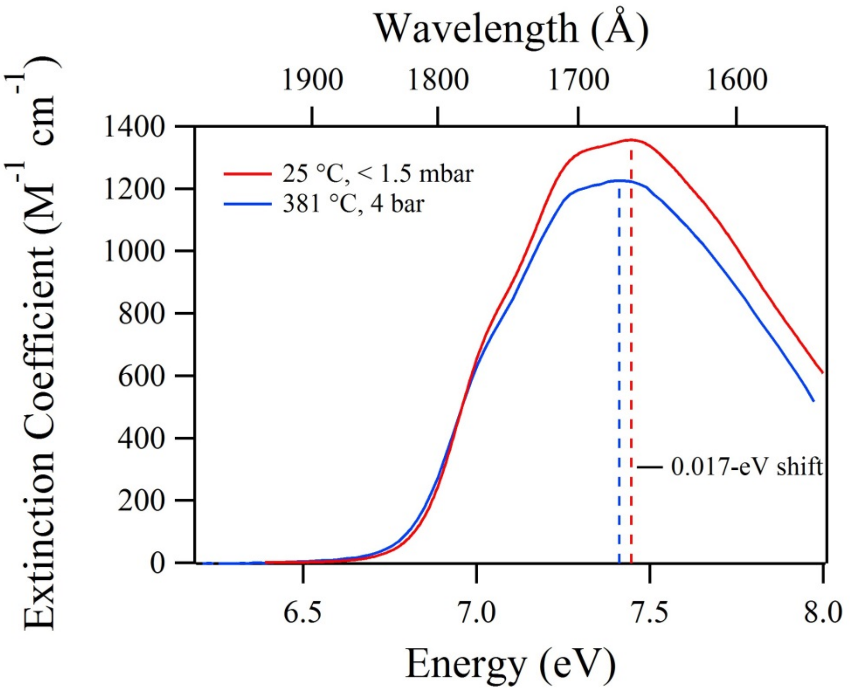

Supplementary Figure 3 | Water gas-phase spectrum vs. SCW low-density spectrum. The 25-°C water gas-phase VUV absorption spectrum[3] (red line) is shown alongside the 381-°C SCW low-pressure absorption spectrum (blue line). Note that the exact shape and width of the gas-phase spectrum are almost entirely reproduced. There is a slight red shift for the SCW spectrum, which we attribute to the influence of excited rotational states.

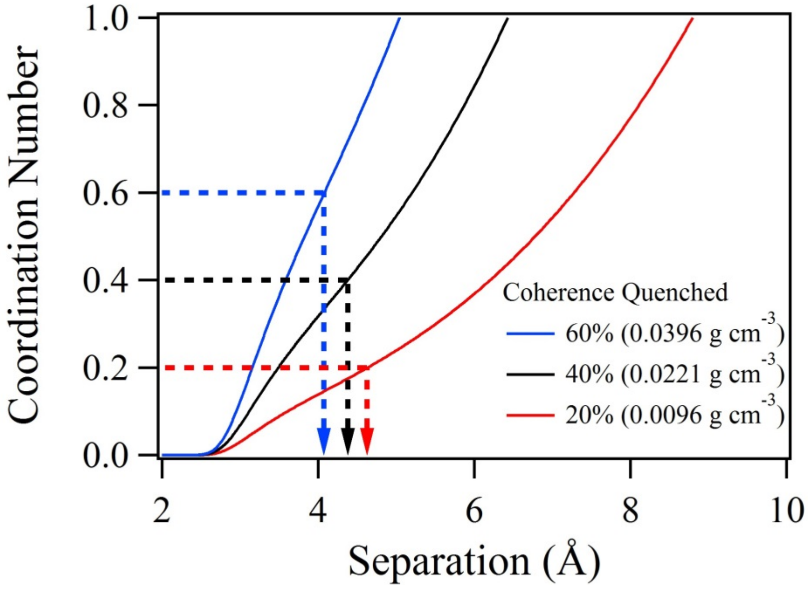

Supplementary Figure 4 | Water dimer running coordination number. The running coordination number vs. intermolecular separation is shown for water dimer at selected densities, based on the work of Jankowski, *et al*.[4] The amount of vibrational coherence observed to be quenched at each density is indicated in the legend. The dashed lines with arrows indicate the critical separation distance R_c necessary at each density to provide the extent of observed quenched coherence. We note that a relatively long intermolecular separation of ca. 4.5 Å is sufficient to remove 20% of the coherence, and hence significantly perturb the electronic structure.

Supplementary Note 1: Gas-Phase Water Monomer Vibronic Structure

The VUV absorption spectrum of gas-phase water shows clear vibronic structure on the low-energy edge. The calculated $\tilde{A}$ state monomer surface is purely dissociative with respect to extension of either O-H bond, and Franck-Condon excitation to this potential energy surface accesses the saddle point above and between the two equivalent dissociation channels. While the $\tilde{A}$ state is dissociative to H and OH product in the asymmetric stretch coordinate, it is still bound in the symmetric stretch coordinate. A remnant of the symmetric stretch resonance is present as some of the trajectories launched on the $\tilde{A}$ state surface do return to their origin once before coupling into the asymmetric stretch dissociative continuum.[5] The structure in the measured absorption spectrum has been assigned to the motion in this saddle point region perpendicular to the dissociation coordinate, corresponding to the symmetric stretch.[5-13] The corresponding frequency is ca. 1850 cm^{-1}.[14] The diffuse nature of the structure reflects the narrowness of the saddle point region, and the consequent short-lived nature of the stretching resonances.

Supplementary Note 2: Lack of Water VUV Absorption Data

The high concentration of pure liquid water (55.3 M at SATP) and a sizable maximum molar extinction coefficient of $\varepsilon_{max} = 1600$ M^{-1} cm^{-1} ($\sigma_{max} = 6.12 \times 10^{-18}$ cm^2 $molecule^{-1}$) at 1500 Å dictate an extremely large Beer-Lambert absorbance for typical optical pathlengths (optical density of ca. 9 even for a very short 1-μm pathlength), greatly inhibiting the convenience of traditional transmission experiments. To overcome this problem, Ikehata, *et al.* recently developed a desktop VUV spectrometer equipped with an attenuated total reflection (ATR) probe.[15] This work demonstrated that an evanescent wave with an ATR geometry achieves a sub-μm path length in water, and therefore VUV spectra were obtained over the entire $\tilde{A}^1B_1 \leftarrow \tilde{X}^1A_1$

absorption band of light and heavy liquid water over the temperature range 10-70 °C. Their results reconfirmed the previously observed spectral red shift with increasing temperature.[16] This work aside, to our knowledge no other recent work directly investigates the first continuum VUV band.

Supplementary Note 3: Subcritical Water Integrated Absorption and Oscillator Strength

The integral of the fitted spectra over all energies (0-∞ eV) is seen to decrease linearly by 0.086% $°C^{-1}$ over the entire temperature range studied (Supplementary Fig. 2). It has been well-documented that in condensed phase the oscillator strength is proportional to the integrated absorption multiplied by a local field correction factor that depends on the refractive index of the medium.[17] While multiple field models exist, for Lorentz, Onsager, or Onsager-Böttcher fields the correction factor decreases with increasing refractive index. Considering the relatively constant spectral shape with increasing temperature encountered in the current data, to preserve oscillator strength we suggest that this trend would have to be accounted for by a corresponding decrease in the water refractive index. To our knowledge, the water refractive index has not been measured above 70 °C[15] in this photon energy regime, so we are unable to provide an accurate in-depth analysis of the water oscillator strength as a function of temperature.

Supplementary Note 4: Extent of H Bonding in Subcritical Water

The weakening of the water H-bond network at elevated temperatures due to added thermal energy has been previously suggested by many studies and reviews.[18-30] This prevents delocalization of the exciton,[31-33] gradually diminishing the spectral energy shift with respect to the gas phase. Assigning the exact number of H bonds per molecule in condensed-phase water

continues to be a hot topic of dispute. Arguments over the relative strength of donor vs. acceptor H-bond moieties, the relative fractions of molecules that display two vs. four H bonds at any instant, and the need for these ideas to be considered within the context of time-averaged long-range structure have been presented by numerous authors, and much of this conversation has been summarized by Chaplin[34] and references therein. Although a mixture of four-linked and two-linked coordination can be fitted well to x-ray absorption data,[35-38] tetrahedral coordination seems to be the most accepted model at this time.[39] For these reasons we make our arguments regarding the extent of H bonding in subcritical water in the context of a traditional tetrahedral coordination picture.

Supplementary Note 5: Low-Density SCW and Gas-Phase Water Peak Energy Discrepancy

The discrepancy in the peak position for the known gas-phase water spectrum[3] and our lowest-density SCW spectrum acquired at 381 °C is real, and to explain it one might consider the contributions of excited vibrational or rotational states arising from the elevated temperature. Vibrational hot bands arising from higher vibrational quantum states would increase the vibronic contributions on low-energy side of the spectrum, causing spectral broadening and possibly a small red shift. However, the Boltzmann populations for the relatively high-frequency water vibrations, whether gas-phase (1595, 3657, 3756 cm^{-1})[40] or liquid-phase (1645, 3277, 3490 cm^{-1}),[34] are not large enough at 381 °C to alter the electronic spectrum within experimental error limits; even the lowest bending vibration in liquid water at 1645 cm^{-1} only has 2.6% population in its first excited level $v = 1$ at this temperature. It is non-trivial to predict specific rovibronic contributions for an asymmetrical top molecule such as water, and we have not attempted this exercise. Regardless, multiple sources indicate that it would indeed be reasonable to find a

spectral shift in a range similar to that reported here, even up to 0.06 eV, due to the influence of ground state rotational excitation.[5-13,41,42] The high rotational excitation of water at 381 °C can actually change the equilibrium ground-state geometry due to centrifugal force, and slightly change the Franck-Condon overlap with the excited surface.

Supplementary Note 6: Past Studies Regarding the Extent and Nature of H Bonding in SCW

Clearly there is disagreement in the literature regarding the extent and nature of H bonding in SCW. Aside from the studies already mentioned, Wernet, *et al.*,[43] claimed 35% monomer species at 380 °C and 300 bar, with H-bonded patches containing 5-10 molecules each based on the results of x-ray Raman scattering experiments. In a series of NMR experiments on SCW, Matubayasi, *et al.*,[30,44] claimed that there is more than one H bond per molecule in SCW in the high-density domain. Other x-ray scattering experiments and molecular dynamics studies by Sit, *et al.*,[45] claimed the dominance of monomers in SCW up to 300 bar and 397 °C, but with significant residual dimer and trimer. Neutron diffraction experiments performed by Otomo, *et al.*,[46] demonstrated coordination numbers of 2.6 molecules for SCW at 263 bar and 400 °C, with few monomers present. Earlier neutron diffraction experiments by Postorino, *et al.*, demonstrated almost no H bonds at 400 °C even at pressures as high as 800 bar.[20,47] These data and more were later examined in detail with new site-site correlation functions by Soper, *et al.*,[48,49] who found approximately 1.5 H bonds per water molecule under these conditions. Other recent Raman work by Sun, *et al.*,[50] demonstrated gas-like behavior for SCW up to densities of 0.11 g cm^{-3}. They suggested that the structure of SCW can be divided into three-dimensional and chain (or string) H-bonded networks, which respectively correspond to liquid- and gas-like SCW, and the critical

region signifies the structural transition between the two domains. Yet more Raman studies by Sahle, *et al.*,[51] indicate approximately 1 H bond per molecule for SCW at 400 °C and 480 bar.

Supplementary Note 7: Critical Distance for Coherence Quenching in SCW

Assuming ideal gas behavior and completely random distances, the probability P_i to find i water molecules within distance R_c of another will be given by Poisson statistics as

$$P_i = \frac{N_s^{\,i}}{i!} e^{-N_s} \quad (1)$$

where given the average number density, ρ, the average number of water molecules within the spherical critical volume, N_s, is given by

$$N_s = \frac{4\pi R_\mathrm{c}^{\,3}\rho}{3} \quad (2)$$

In order to observe the vibrational progression, we need to have zero water molecules within R_c, so the necessary equation reduces to $P_0 = e^{-N_s}$, and a simple exponential behavior with increasing density is recovered. By this analysis, R_c must be 5.49 Å based on Fig. 7a, where we used a density of 0.0432 g cm^{-3}, corresponding to the point where the coherence feature has been reduced to 1/*e* of its maximum value.

The assumption of ideal gas behavior is highly questionable in the preceding analysis, so we make an additional estimate based on the radial distribution function $g(r)$ calculated for dimers in the limit of low water density. Assuming that trimers and higher clusters are absent, we can define

$$g(r) = e^{-V_\mathrm{ww}(r)/k_\mathrm{B}T} \quad (3)$$

where the potential of mean force V_{ww} is just the high quality water-water dimer potential proposed by Jankowski and coworkers,[4] with an average taken over all possible angles of approach for center-of-mass separation, r. The running coordination number is defined by

$$N_c(R) = 4\pi\rho \int_0^R e^{-V_{\mathrm{ww}}(r)/k_{\mathrm{B}}T} r^2 dr \tag{4}$$

and represents the average number of water molecules found within distance R of a water molecule at the origin. The running coordination number is plotted for several densities in Supplementary Fig. 4, from the dimer $g(r)$ function. It must also be true that

$$N_c(R) = 0(P_0) + 1(P_1) + 2(P_2) + \dots \tag{5}$$

where the P_i represent normalized probability for finding i molecules within distance R. In the limit of low density we can truncate the sum after P_1, in which case $N_c(R) = P_1$. Again based on Fig. 7a, 20% of the vibrational structure has been quenched at a density of 0.0096 g cm^{-3}. We assume this means $P_1 = N_c = 0.20$ at this density. From the plot in Supplementary Fig. 4, we find this equality to be true for a critical distance $R_{\mathrm{c}} = 4.63$ Å. Similarly, quenching of 40% or 60% of the coherence feature gives $R_{\mathrm{c}} = 4.37$ and 4.08 Å, respectively. As expected, these numbers are smaller than the ideal gas estimate of $R_{\mathrm{c}} = 5.49$ Å. More importantly, all of these values are significantly larger than the experimentally known equilibrated dimer separation of 2.98 Å.[52]

Supplementary References

1 Kröckel, L. & Schmidt, M. A. Extinction properties of ultrapure water down to deep ultraviolet wavelengths. *Opt. Mater. Express* **4**, 1932-1942, doi:10.1364/OME.4.001932 (2014).

2 Tretyakov, M. Y., Serov, E. A. & Odintsova, T. A. Equilibrium thermodynamic state of water vapor and the collisional interaction of molecules. *Radiophys. Quantum Electron.* **54**, 700-716, doi:10.1007/s11141-012-9332-x (2012).

3 Mota, R. *et al.* Water VUV electronic state spectroscopy by synchrotron radiation. *Chem. Phys. Lett.* **416**, 152-159, doi:10.1016/j.cplett.2005.09.073 (2005).

4 Jankowski, P. *et al.* Ab Initio Water Pair Potential with Flexible Monomers. *J. Phys. Chem. A* **119**, 2940-2964, doi:10.1021/jp512847z (2015).

5 Engel, V. *et al.* Photodissociation of water in the first absorption band: a prototype for dissociation on a repulsive potential energy surface. *J. Phys. Chem.* **96**, 3201-3213, doi:10.1021/j100187a007 (1992).

6 Engel, V., Meijer, G., Bath, A., Andresen, P. & Schinke, R. The $\tilde{C}\rightarrow\tilde{A}$ emission in water: Theory and experiment. *J. Chem. Phys.* **87**, 4310-4314, doi:10.1063/1.452889 (1987).

7 Engel, V. & Schinke, R. Isotope effects in the fragmentation of water: The photodissociation of HOD in the first absorption band. *J. Chem. Phys.* **88**, 6831-6837, doi:10.1063/1.454381 (1988).

8 Engel, V., Schinke, R. & Staemmler, V. An ab initio calculation of the absorption cross section of water in the first absorption continuum. *Chem. Phys. Lett.* **130**, 413-418, doi:10.1016/0009-2614(86)80496-1 (1986).

9 Engel, V., Schinke, R. & Staemmler, V. Photodissociation dynamics of H_2O and D_2O in the first absorption band: A complete *ab initio* treatment. *J. Chem. Phys.* **88**, 129-148, doi:10.1063/1.454645 (1988).

10 Hennig, S., Engel, V., Schinke, R. & Staemmler, V. Emission spectroscopy of photodissociating water molecules: A time-independent ab initio study. *Chem. Phys. Lett.* **149**, 455-462, doi:10.1016/0009-2614(88)80363-4 (1988).

11 Schinke, R. & Engel, V. The rotational reflection principle in photodissociation dynamics. *Faraday Discuss.* **82**, 111-124, doi:10.1039/dc9868200111 (1986).

12 Schinke, R., Engel, V. & Staemmler, V. Rotational state distributions in the photolysis of water: Influence of the potential anisotropy. *J. Chem. Phys.* **83**, 4522-4533, doi:10.1063/1.449021 (1985).

13 Schinke, R., Weide, K., Heumann, B. & Engel, V. Diffuse structures and periodic orbits in the photodissociation of small polyatomic molecules. *Faraday Discuss.* **91**, 31-46, doi:10.1039/dc9919100031 (1991).

14 Wang, H. t., Felps, W. S. & McGlynn, S. P. Molecular Rydberg states. VII. Water. *J. Chem. Phys.* **67**, 2614-2628, doi:10.1063/1.435173 (1977).

15 Ikehata, A., Higashi, N. & Ozaki, Y. Direct observation of the absorption bands of the first electronic transition in liquid H_2O and D_2O by attenuated total reflectance far-UV spectroscopy. *J. Chem. Phys.* **129**, doi:10.1063/1.3039080 (2008).

16 Marin, T. W., Takahashi, K. & Bartels, D. M. Temperature and density dependence of the light and heavy water ultraviolet absorption edge. *J. Chem. Phys.* **125**, doi:10.1063/1.2338521 (2006).

17 Iweibo, I., Obi-Egbedi, N. O., Chongwain, P. T., Lesi, A. F. & Abe, T. The theory of electronic intensity in solution or condensed media. *J. Chem. Phys.* **93**, 2238-2245, doi:10.1063/1.459057 (1990).

18 Nilsson, A. & Pettersson, L. G. M. The structural origin of anomalous properties of liquid water. *Nat. Commun.* **6**, 8998, doi:10.1038/ncomms9998 (2015).

19 Ratcliffe, C. I. & Irish, D. E. Vibrational spectral studies of solutions at elevated temperatures and pressures. 5. Raman studies of liquid water up to 300.degree.C. *J. Phys. Chem.* **86**, 4897-4905, doi:10.1021/j100222a013 (1982).

20 Postorino, P., Tromp, R. H., Ricci, M. A., Soper, A. K. & Neilson, G. W. The interatomic structure of water at supercritical temperatures. *Nature* **366**, 668-670, doi:10.1038/366668a0 (1993).

21 Carey, D. M. & Korenowski, G. M. Measurement of the Raman spectrum of liquid water. *J. Chem. Phys.* **108**, 2669-2675, doi:10.1063/1.475659 (1998).

22 Mizan, T. I., Savage, P. E. & Ziff, R. M. Temperature Dependence of Hydrogen Bonding in Supercritical Water. *J. Phys. Chem.* **100**, 403-408, doi:10.1021/jp951561t (1996).

23 Jedlovszky, P. *et al.* Analysis of the hydrogen-bonded structure of water from ambient to supercritical conditions. *J. Chem. Phys.* **108**, 8528-8540, doi:10.1063/1.476282 (1998).

24 Boero, M., Terakura, K., Ikeshoji, T., Liew, C. C. & Parrinello, M. Water at supercritical conditions: A first principles study. *J. Chem. Phys.* **115**, 2219-2227, doi:10.1063/1.1379767 (2001).

25 Cabral do Couto, P. & Chipman, D. M. Insights into the ultraviolet spectrum of liquid water from model calculations. *J. Chem. Phys.* **132**, 244307, doi:10.1063/1.3453248 (2010).

26 Antipova, M. L., Gurina, D. L. & Petrenko, V. E. The structure of H-bonded clusters in sub- and supercritical water. *Russ. J. Phys. Chem. A* **85**, 977-982, doi:10.1134/s0036024411060045 (2011).

27 Šašić, S., Segtnan, V. H. & Ozaki, Y. Self-Modeling Curve Resolution Study of Temperature-Dependent Near-Infrared Spectra of Water and the Investigation of Water Structure. *J. Phys. Chem. A* **106**, 760-766, doi:10.1021/jp013436p (2002).

28 Krynicki, K., Green, C. D. & Sawyer, D. W. Pressure and temperature dependence of self-diffusion in water. *Farad. Discuss.* **66**, 199-208, doi:10.1039/DC9786600199 (1978).

29 Hoffmann, M. M. & Conradi, M. S. Are There Hydrogen Bonds in Supercritical Water? *J. Am. Chem. Soc.* **119**, 3811-3817, doi:10.1021/ja964331g (1997).

30 Matubayasi, N., Wakai, C. & Nakahara, M. Structural study of supercritical water. I. Nuclear magnetic resonance spectroscopy. *J. Chem. Phys.* **107**, 9133-9140, doi:10.1063/1.475205 (1997).

31 Brancato, G., Rega, N. & Barone, V. Accurate Density Functional Calculations of Near-Edge X-Ray and Optical Absorption Spectra of Liquid Water Using Nonperiodic Boundary Conditions: The Role of Self-Interaction and Long-Range Effects. *Phys. Rev. Lett.* **100**, 107401, doi:10.1103/PhysRevLett.100.107401 (2008).

32 Garbuio, V., Cascella, M. & Pulci, O. Excited state properties of liquid water. *J. Phys.: Condens. Matter* **21**, 033101, doi:10.1088/0953-8984/21/3/033101 (2009).

33 Garbuio, V., Cascella, M., Reining, L., Sole, R. D. & Pulci, O. *Ab Initio* Calculation of Optical Spectra of Liquids: Many-Body Effects in the Electronic Excitations of Water. *Phys. Rev. Lett.* **97**, 137402, doi:10.1103/PhysRevLett.97.137402 (2006).

34 Chaplin, M. *Water Structure and Science*, http://www1.lsbu.ac.uk/water/water_structure_science.html (2017).

35 Leetmaa, M. *et al.* Diffraction and IR/Raman data do not prove tetrahedral water. *J. Chem. Phys.* **129**, 084502, doi:10.1063/1.2968550 (2008).

36 Huang, C. *et al.* The inhomogeneous structure of water at ambient conditions. *Proc. Natl. Acad. Sci. U.S.A.* **106**, 15214-15218, doi:10.1073/pnas.0904743106 (2009).

37 Myneni, S. *et al.* Spectroscopic probing of local hydrogen-bonding structures in liquid water. *J. Phys.: Condens. Matter* **14**, L213, doi:10.1088/0953-8984/14/8/106 (2002).

38 Wernet, P. *et al.* The Structure of the First Coordination Shell in Liquid Water. *Science* **304**, 995-999, doi:10.1126/science.1096205 (2004).

39 Kühne, T. D. & Khaliullin, R. Z. Nature of the Asymmetry in the Hydrogen-Bond Networks of Hexagonal Ice and Liquid Water. *J. Am. Chem. Soc.* **136**, 3395-3399, doi:10.1021/ja411161a (2014).

40 Tennyson, J. *et al.* IUPAC critical evaluation of the rotational–vibrational spectra of water vapor, Part III: Energy levels and transition wavenumbers for H216O. *J. Quant. Spectrosc. Radiat. Transfer* **117**, 29-58, doi:10.1016/j.jqsrt.2012.10.002 (2013).

41 Schinke, R., Engle, V., Andresen, P., Hausler, D. & Balintkurti, G. G. Photodissociation of single H_2O quantum states in the first absorption band: Complete characterization of OH rotational and Lambda-doublet state distributions. *Phys. Rev. Lett.* **55**, 1180-1183, doi:10.1103/PhysRevLett.55.1180 (1985).

42 Herzberg, G. *Molecular spectra and molecular structure. III. Electronic spectra and electronic structure of polyatomic molecules.* (Van Nostrand, 1966).

43 Wernet, P. *et al.* Spectroscopic characterization of microscopic hydrogen-bonding disparities in supercritical water. *J. Chem. Phys.* **123**, doi:10.1063/1.2064867 (2005).

44 Matubayasi, N., Wakai, C. & Nakahara, M. Structural study of supercritical water. II. Computer simulations. *J. Chem. Phys.* **110**, 8000-8011, doi:10.1063/1.478728 (1999).

45 Sit, P. H. L. *et al.* Hydrogen bonding and coordination in normal and supercritical water from x-ray inelastic scattering. *Phys. Rev. B* **76**, doi:10.1103/PhysRevB.76.245413 (2007).

46 Otomo, T. *et al.* Partial pair correlation functions of low-density Supercritical water determined by neutron diffraction with the H/D isotopic substitution method. *J. Phys. Chem. B* **112**, 4687-4693, doi:10.1021/jp711434n (2008).

47 Tromp, R. H., Postorino, P., Neilson, G. W., Ricci, M. A. & Soper, A. K. Neutron diffraction studies of H_2O/D_2O at supercritical temperatures. A direct determination of g_{HH}(r), g_{OH}(r), and g_{OO}(r). *J. Chem. Phys.* **101**, 6210-6215, doi:10.1063/1.468403 (1994).

48 Soper, A. K. The radial distribution functions of water and ice from 220 to 673 K and at pressures up to 400 MPa. *Chem. Phys.* **258**, 121-137, doi:10.1016/s0301-0104(00)00179-8 (2000).

49 Soper, A. K., Bruni, F. & Ricci, M. A. Site-site pair correlation functions of water from 25 to 400 degrees C: Revised analysis of new and old diffraction data. *J. Chem. Phys.* **106**, 247-254, doi:10.1063/1.473030 (1997).

50 Sun, Q. & Wang, Q. Hydrogen Bonded Networks in Supercritical Water. *J. Phys. Chem. B* **118**, 11253-11258, doi:10.1021/jp503474s (2014).

51 Sahle, C. J. *et al.* Microscopic structure of water at elevated pressures and temperatures. *Proc. Natl. Acad. Sci. U.S.A.* **110**, 6301-6306, doi:10.1073/pnas.1220301110 (2013).

52 Odutola, J. A. & Dyke, T. R. Partially deuterated water dimers: Microwave spectra and structure. *J. Chem. Phys.* **72**, 5062-5070, doi:10.1063/1.439795 (1980).
